# Neuromuscular Monitoring, Muscle Relaxant Use, and Reversal at a Tertiary Teaching Hospital 2.5 Years after Introduction of Sugammadex: Changes in Opinions and Clinical Practice

**DOI:** 10.1155/2015/367937

**Published:** 2015-01-22

**Authors:** Thomas Ledowski, Jing Shen Ong, Tom Flett

**Affiliations:** ^1^Department of Anaesthesia, Royal Perth Hospital, Perth, WA 6000, Australia; ^2^University of Western Australia, Perth, WA 6009, Australia; ^3^Royal Perth Hospital, Perth, WA 6000, Australia; ^4^Department of Intensive Care, The Alfred Hospital, Melbourne, VIC 3000, Australia

## Abstract

Sugammadex was introduced to Royal Perth Hospital in early 2011 without access restriction. Two departmental audits (26-page online survey and 1-week in-theatre snapshot audit) were undertaken to investigate the change of beliefs and clinical practice related to the use of neuromuscular blocking agents at the Royal Perth Hospital since this introduction. Results were compared with data from 2011. We found that, in the 2.5 years since introduction of Sugammadex, more anesthetists (69.5 versus 38%) utilized neuromuscular monitoring, and aminosteroidal neuromuscular blocking agents were used in 94.3% of cases (versus 77% in 2011). Furthermore, 53% of anesthetists identified with a practice of “deeper and longer” intraoperative paralysis of patients. All 71 patients observed during the 5-day in-theatre audit were reversed with Sugammadex. Since the introduction of Sugammadex, 69% (*n* = 20) of respondents felt it provided “faster turnover,” less postoperative residual neuromuscular blockade (*n* = 23; 79%), and higher anesthetist satisfaction (*n* = 17; 59%). 45% (*n* = 13) of colleagues reported that they would feel professionally impaired without the unrestricted availability of Sugammadex, and 1 colleague would refuse to work in a hospital without this drug being freely available. In clinical practice Sugammadex was frequently (57%) mildly overdosed, with 200 mg being the most commonly administered dose.

## 1. Introduction

Sugammadex was introduced to Royal Perth Hospital (RPH) in early 2011 without access restriction. Since early 2011, its use has increased to approximately 7000 doses (200 mg) per year in 2013. Two previously published audits [1, 2] comparing the “pre-” and “post-”Sugammadex practice of neuromuscular blocking agent (NMBA) use and reversal in 2011 identified an approximate 50% decline in the use of neostigmine since introduction of Sugammadex. They also revealed a low rate of neuromuscular monitoring (38%) and, correspondingly, a very high incidence of postoperative residual neuromuscular blockade (RNMB) and associated complications.

The aforementioned investigations investigated changes in anesthesia practice and patient postoperative outcome within only a few months from the introduction of Sugammadex. In contrast to studying the status quo as well as “short term” changes, it was the aim of the current audit to investigate whether the introduction of Sugammadex has resulted in a long term (2 years) change of anesthetists' NMBA associated practice and beliefs.

## 2. Methods

Both projects were approved by the RPH Department of Quality and Safety as clinical audits. Firstly, a 26-page web-based (*Survey Monkey)* questionnaire (see full survey in Supplementary Material available online at http://dx.doi.org/10.1155/2015/367937) asking questions around the matter of NMBA and reversal use was sent to all RPH anesthetists in mid-2013. Secondly, a one-week (Monday to Friday; 8 a.m.–5 p.m.) prospective “snapshot” audit was performed within RPH theatres in October 2013. The latter included all noncardiothoracic patients receiving NMBA at RPH during the specified time. This project aimed to gather information about the practice of neuromuscular monitoring, NMBA, and reversal use and the incidence of RNMB. Data for this “in-theatre” audit was gathered by a research assistant present during the phase of patients' tracheal extubation. However, the decision whether or not to monitor or reverse RNMB was entirely left to the attending anesthetist. If neuromuscular monitoring was applied, the kinemyometric monitoring (KMG; quantitative monitoring) module (GE Healthcare, Helsinki, Finland) was used.

Wherever meaningful, and in order to achieve a longitudinal view of changes in anesthesia practice, results from both audits were compared with those of the two similar projects published by us previously [1, 2].

## 3. Results

### 3.1. Online Survey

Twenty-four consultants as well as 14 registrars replied to the online survey, resulting in an overall response rate of 32 percent.

#### 3.1.1. NMBA Use

72% (*n* = 26) of colleagues replied that they would use muscle relaxants more often than in 2011 and stated “optimizing anesthesia and surgery” as the main reason for doing so. 54.3% (*n* = 19) specifically stated that they paralyze patients intraoperatively deeper and for longer, with 65% (*n* = 24) of all respondents believing that doing so may improve surgical conditions without the need to increase the depth of anesthesia. However, only 25.8% (*n* = 8) of the above-mentioned 65% (*n* = 24) stated that they had actually seen such benefits in their own practice. The remaining respondents did see a potential benefit, but without this being evident in their daily life. Interestingly, and uninfluenced by the introduction of Sugammadex, more than 90% of respondents did not rate Succinylcholine as a superseded drug for rapid sequence induction.

#### 3.1.2. Monitoring

Neuromuscular monitoring was stated to be the single most important instrument to make a decision about NMBA reversal by the majority (*n* = 22) of respondents. Only a few respondents preferred timing (*n* = 1), clinical evidence (*n* = 2), and type of NMBA based reversal (*n* = 1).

#### 3.1.3. Reversal

Overall, reversal rates were relatively high with 48% (*n* = 12) respondents stating that they practice NMBA reversal in 76–100% of general anesthetics and 31% (*n* = 8) respondents reversing NMBA in 51–75% cases. 55% (*n* = 21) of respondents stated that they use reversal agents more often compared to 2011.

Interestingly, and despite making Sugammadex available without restriction in 2011, many (41%; *n* = 16) respondents reported only having used the drug “routinely” since 2012.

Sugammadex was chosen in more than 75% of reversal cases by 73.4% (*n* = 27) of respondents. Though 86% (*n* = 25) of respondents stated using a nerve stimulator to determine need and dose for Sugammadex-based reversal, a relatively high proportion (41%; *n* = 12) stated not using such methods to check the success of the reversal due to the high reliability of the drug.

In the context of Sugammadex reversal, personal experiences of faster “case turnover,” less postoperative residual neuromuscular blockade, and higher anaesthetist satisfaction were quoted by 69% (*n* = 20), 79% (*n* = 23), and 59% (*n* = 17), respectively. 45% (*n* = 13) of colleagues reported that they would feel professionally impaired without the unrestricted availability of Sugammadex, and 1 colleague would even refuse to work in a hospital without this drug being freely available. In clinical practice Sugammadex was frequently (57%) mildly overdosed (based on the official prescription information), with 200 mg being the most commonly administered dose. 68% of respondents stated knowing the price of Sugammadex to the RPH anaesthesia department, but only 43% saw this as an important factor influencing their practice.

### 3.2. In-Theatre Audit

Data of 71 patients (52 ± 18 (16–85) years) were analyzed.

#### 3.2.1. NMBA Use

NMBA were used during plastic surgery (19.7%), general surgery (23.9%), orthopedic surgery (16.9%), or other surgical specialty procedures (39.4%). Rocuronium was used for intubation in 88.7% of patients, Vecuronium in 5.6%, Succinylcholine in 2.8%, and Cisatracurium and Mivacurium in 1.4% of cases. A second dose of NMBA was given in 20 patients, with Rocuronium chosen in 19 of those.

#### 3.2.2. Monitoring

The need for reversal was determined using clinical signs only in 30.5%, whereas in 69.5% of patients neuromuscular monitoring was used. Train of four (TOF) “fade” was detected at the end of surgery in 38 patients (53.3%).

#### 3.2.3. Reversal

Sugammadex was administered in all 38 patients in whom a TOF fade had been detected and in 20 patients in whom either no fade had been found (*n* = 2) or no monitoring had been used (*n* = 18). Remarkably, no Neostigmine or other cholinesterase-inhibitor was used at all (despite introduction of Sugammadex in 2011, the choice of reversal agent is fully at the discretion of the attending anesthesiologist). Rated by the official prescription information (PI) for Sugammadex the drug was slightly overdosed in 33 and underdosed in 7 cases. Main reasons for incorrect dosing included the desire not to waste Sugammadex, personal dosing experience that differed from the official PI, and the lack of any monitoring available. The doses administered were documented correctly in the vast majority of anesthetics (94.2%). However, the use and result of neuromuscular monitoring were only documented with sufficient detail in about half of the patients.

## 4. Discussion

2.5 years after the introduction of Sugammadex without access restriction our results support that the vast majority of RPH anaesthetists were choosing an aminosteroidal NMBA (94.3%), Sugammadex (100%) combination when paralyzing patients. This usage pattern constitutes a significant increase of aminosteroidal NMBA use from 61% (2010) [1] and 84% (2011) [1], as well as a very large increment in the use of Sugammadex (usage in 2010: 10%, and usage in 2011: 63% [1]).

65% of the surveyed anesthetists stated their belief that deep and extended neuromuscular blockade may result in better surgical conditions. Despite this, many colleagues stated that although they believed in the benefits of deep neuromuscular blockade, they had not seen such effects in their own practice. Though the evidence for improved surgical conditions by means of muscle relaxation is relatively sparse, more recently, studies have identified measurable benefits [2–6]. In fact, within just one year (2014), various authors [4–6] described significantly improved surgical conditions during laparoscopic surgery under deep (no TOF twitch) versus moderate (TOF 2–4 twitches) neuromuscular blockade. Compared to these seemingly unanimous results it is interesting to find that such changes had only been observed by a minority of anesthetists (25.8%) in this audit. A possible explanation for these observations may be that deep (versus moderate) blockade is not yet routinely practiced by many anesthetists, and secondly these audit reviews did not survey any surgical opinions.

A second hypothetical benefit for using more intraoperative NMBA quoted by the surveyed anesthetists was a potentially improved patient outcome due to a reduced use of hypnotic anesthetic agents resulting in a more appropriate (monitored) depth of anesthesia. Though direct evidence for this point is missing, previous data have linked (deep) anesthesia with impaired patient outcome [7] and a large international multicenter study (Balanced trial; Australian and New Zealand College of Anaesthetists).

A very encouraging result of our audits was that the attitude of anesthetists towards neuromuscular monitoring had significantly improved from April 2011 to October 2013 with only a minority of colleagues (*n* = 1 in the web-based survey) rating clinical signs of adequate neuromuscular recovery as sufficiently reliable tools for clinical practice. The latter rating is surprisingly low compared to a 2010 survey performed by Naguib et al. [8] who identified that 43.5% of European and even 68.2% of US anesthetists believed clinical signs to be sufficiently accurate. However, in reality the sensitivity of various clinical signs (i.e., 5 s leg lift) to detect inadequate neuromuscular recovery has been found to be extremely poor [9].

In the context of neuromuscular monitoring, 43% of surveyed anesthetists stated limiting monitoring to intraoperative (prereversal) use only and omitting using neuromuscular monitors to check the adequacy of Sugammadex-based reversal. Though Sugammadex has been shown to result in significantly lower rates of RNMB [2] when compared to timing of NMBA use or neostigmine, it has also been documented that not monitoring the success of Sugammadex-based reversal was still correlated with an 8–9.4% RNMB rate [2, 10]. Our survey also observed the trend to a “one size fits all” approach in dosing Sugammadex. The most commonly chosen dose of 200 mg frequently constituted a mild overdose and rarely an underdose of the drug. Though there is no direct evidence to link mild over- or underdosage of Sugammadex to undesirable patient outcomes, more severe underdosing could potentially result in recurarization [11] and should hence be avoided. More concerning than the trend to a 200 mg dose for all patients was the fact that 18 patients received Sugammadex without any preceding neuromuscular monitoring. As the results of such monitoring are imperatively important to determine the need as well as the correct dose of the drug, a failure to monitor neuromuscular function may result in unnecessary drug administration or significant dosing errors. The latter not only adds to patient risk and healthcare costs, but also poses a risk for litigation. In the same context anesthetists should also document all neuromuscular monitoring efforts and results, as well as the drugs given to reverse RNMB. The rate of monitoring documentation (approximately 50%) we found in our survey was certainly alarmingly low and needs to drastically improve.

Our “snapshot” in-theatre audit did not directly measure RNMB. However, the high rate of neuromuscular monitoring as well as the fact that all patients in whom fade was detected received Sugammadex for reversal of NMBA effects at least suggests that the overall rate of RNMB at our institution may have decreased from the very high incidences (50–60%) reported by us in 2011 [2].

We conclude that the unrestricted introduction of Sugammadex at our institution has resulted in a near complete shift to a Rocuronium-Sugammadex combination. The trend to use a “one size fits all” dose of Sugammadex has been identified and requires further staff education.

## Supplementary Material

Full survey sent to anesthesiologists via a link to the Survey Monkey website
