# Supplementary material for: Neuromuscular Monitoring, Muscle Relaxant Use, and Reversal at a Tertiary Teaching Hospital 2.5 Years after Introduction of Sugammadex: Changes in Opinions and Clinical Practice
Source: Anesthesiol Res Pract. 2015 Jan 22;2015:367937. doi: 10.1155/2015/367937 (PMC4312631; doi:10.1155/2015/367937)
Supplement: Supplementary file 1 — Full survey sent to anesthesiologists via a link to the Survey Monkey website [file 367937.f1.pdf]

Sugammadex - 2.5 Years On

***1. How many years of Anaesthesia training have you undertaken?**

- ◯ 1
- ◯ 2
- ◯ 3
- ◯ 4
- ◯ 5
- ◯ >5
- ◯ Consultant < 5yrs
- ◯ Consultant 5-10yrs
- ◯ Consultant > 10yrs

***2. When did you first start using Sugammadex "routinely" (not just in emergency scenarios)?**

- ◯ 2010
- ◯ 2011
- ◯ 2012
- ◯ I don't routinely use Sugammadex

***3. Do you always reverse Non Depolarising Neuromuscular Blocking Agents (NMBA)?**

- ◯ Yes always
- ◯ Not always

***4. Do you reverse NMBA more often since introduction of sugammadex at RPH?**

- ◯ Yes, I reverse more often
- ◯ No, I use less reversal
- ◯ About the same

***5. After NMBA use, how often do you use reversal agents (neostigmine or sugammadex)?**

- ◯ 0%
- ◯ 1-25%
- ◯ 26-50%
- ◯ 51-75%
- ◯ 76-100%

***6. Which of the following factors help you in making the decision to administer reversal? Check all that apply**

- ☐ Type of NMBA
- ☐ Total dose of NMBA
- ☐ Timing of the last NMBA dose
- ☐ Qualitative neuromuscular monitoring (conventional nerve stimulator)
- ☐ Quantitative neuromuscular monitoring (numeric assesment TOF ratio)
- ☐ Clinical evidence
- ☐ Other (please specify)

***7. In your personal clinical practice, which of the following factors is the single most important in making the decision to administer reversal?**

- ◯ Type of NMBA
- ◯ Total dose of NMBA
- ◯ Timing of the last NMBA dose
- ◯ Qualitative neuromuscular monitoring (conventional nerve stimulator)
- ◯ Quantitative neuromuscular monitoring (numeric assesment TOF ratio)
- ◯ Clinical evidence
- ◯ Other (please specify)

***8. If administering a reversal agent, please specify the percentage use of Sugammadex**

- ◯ 0%
- ◯ 1-25%
- ◯ 26-50%
- ◯ 51-75%
- ◯ 76-99%
- ◯ 100%

***9. Based on your clinical experience at RPH, what subgroups of patients are best suited for reversal with Sugammadex?**

[ ]

10. Do you use NMBA more often since unrestricted introduction of Sugammadex at RPH?

- ☐ Yes, to optimise Anaesthesia
- ☐ Yes, to optimise Surgery
- ☐ No, my practice has not changed
- ☐ No, I use less NMBA

11. Has your choice of NMBA changed since unrestricted introduction of Sugammadex at RPH?

- ◯ Yes I use more Aminosteroidal NMBA
- ◯ No
- ◯ Other (please specify)

[ ]

***12. Intraoperatively, do you paralyze patients deeper or for longer since unrestricted introduction of Sugammadex at RPH?**

- ◯ Yes
- ◯ No

13. Do you believe that deeper neuromuscular paralysis may be a way to safely improve surgical conditions with the chance to reduce the use of volatiles/opioids (depth of anaesthesia monitoring assumed)?

- ◯ Yes, I believe deeper paralysis may improve surgical conditions and patient outcome (no need for too deep and potentially detrimental [e.g. BIS < 40] anaesthetic; faster recovery).
- ◯ No, I do not believe that deep paralysis can safely achieve better surgical conditions and/or improved patient outcome.

***14. As you see potential benefits of deep paralysis: Is this your own clinical practice?**

- ◯ Yes, I do practice the concept of deep paralysis
- ◯ No, although I see a hypothetical benefit, I do not practice the concept of deep paralysis

***15. Have you personally experienced any of the following since unrestricted introduction of sugammadex at RPH (check all that apply)?**

- ☐ Faster patient turnover
- ☐ Less PONV in recovery
- ☐ Surgeons more satisfied with anaesthesia
- ☐ Anaesthetist more satisfied with anaesthesia
- ☐ Faster surgery
- ☐ Lower incidence of residual paralysis
- ☐ Patient outcome improved in recovery (better oxygen saturation, patient "more awake")
- ☐ Sugammadex related side effects (-please specify in 'Other')
- ☐ Problems with re-intubation of patient after use of sugammadex (aminosteroid NMBA no longer usable)
- ☐ Other (please specify)

____

***16. Do you use a higher dose of NMBA for the purpose of a 'modified RSI' since unrestricted introduction of Sugammadex at RPH?**

- ◯ Yes, for Rocuronium
- ◯ Yes, for Vecuronium
- ◯ Yes, for both Vecuronium and Rocuronium
- ◯ No

If YES, dose (mg/kg) of what NMBA?

____

***17. Do you consider Suxamethonium a 'superseded' drug since unrestricted introduction of Sugammadex at RPH?**

- ◯ Yes
- ◯ No

***18. When using sugammadex at the end of anaesthesia: Do you frequently (ie > 25%) use more than a 200 mg vial per patient?**

- ◯ No
- ◯ > 25% of cases
- ◯ > 50% of cases
- ◯ > 75% of cases

***19. Have you ever used a 16mg/kg dose of Sugammadex?**

- ◯ Yes
- ◯ No

If YES, how many times?

[ ]

***20. In your view, at what Train of Four count would 2mg/kg Sugammadex produce rapid and reliable reversal of aminosteroidal NMBA?**

- ◯ 0/4
- ◯ 1/4
- ◯ 2/4
- ◯ 3/4
- ◯ 4/4
- ◯ Any Response

***21. Do you routinely use a nerve stimulator prior to administration of Sugammadex?**

- ◯ Yes
- ◯ No

***22. Do you routinely check the success of NMBA reversal after administration of sugammadex?**

- ◯ No, sugammadex is so reliable that in my clinical routine monitoring is redundant
- ◯ yes, I always monitor the success of NMBA reversal prior to extubation

***23. How much time do you allow from time of administration of Sugammadex to extubation?**

- ◯ <1min
- ◯ 1-3min
- ◯ 3-5min
- ◯ 5-10min
- ◯ I don't take notice of the time as it is not clinically significant

***24. Do you know the cost of Sugammadex to the RPH Anaesthesia department?**

- ◯ Yes, and this is an important consideration in my usage of reversal agents
- ◯ Yes, but this is not an important consideration in my usage of reversal agents
- ◯ I do not know the cost

***25. After your experience with sugammadex at RPH - do you feel that you are able to practice anaesthesia as safely (in patients requiring muscle relaxation) at hospitals which do not provide unrestricted use of sugammadex?**

- ☐ Yes, no problem! Neostigmine is as safe as sugammadex.
- ☐ Yes, no problem! Timing is as safe as sugammadex.
- ☐ No, I feel that my anaesthetic is impaired if my access to sugammadex is hindered.
- ☐ No, I would refuse working at such hospital or demand access to sugammadex.

26. Please indicate why you feel less safe without access to sugammadex.
